# Investigation of innate immunity genes *CARD4*, *CARD8 *and *CARD15 *as germline susceptibility factors for colorectal cancer

**DOI:** 10.1186/1471-230X-9-79

**Published:** 2009-10-20

**Authors:** Nikolaus Möckelmann, Witigo von Schönfels, Stephan Buch, Oliver von Kampen, Bence Sipos, Jan Hendrik Egberts, Philip Rosenstiel, Andre Franke, Mario Brosch, Sebastian Hinz, Christian Röder, Holger Kalthoff, Ulrich R Fölsch, Michael Krawczak, Stefan Schreiber, Clemens Dieter Bröring, Jürgen Tepel, Clemens Schafmayer, Jochen Hampe

**Affiliations:** 1 Department of General Internal Medicine Christian-Albrechts-University, Kiel, Germany; 2 POPGEN Biobank project Christian-Albrechts-University, Kiel, Germany; 3 Institute for Pathology Christian-Albrechts-University, Kiel, Germany; 4 Department of General and Thoracic Surgery Christian-Albrechts-University, Kiel, Germany; 5 Institute of Clinical Molecular Biology Christian-Albrechts-University, Kiel, Germany; 6 Institute for Experimental Cancer Research/Comprehensiv Cancer Center North Christian-Albrechts-University, Kiel, Germany; 7 Institute of Medical Informatics and Statistics Christian-Albrechts-University, Kiel, Germany

## Abstract

**Background:**

Variation in genes involved in the innate immune response may play a role in the predisposition to colorectal cancer (CRC). Several polymorphisms of the *CARD15 *gene (caspase activating recruitment domain, member 15) have been reported to be associated with an increased susceptibility to Crohn disease. Since the *CARD15 *gene product and other CARD proteins function in innate immunity, we investigated the impact of germline variation at the *CARD4*, *CARD8 *and *CARD15 *loci on the risk for sporadic CRC, using a large patient sample from Northern Germany.

**Methods:**

A total of 1044 patients who had been operated with sporadic colorectal carcinoma (median age at diagnosis: 59 years) were recruited and compared to 724 sex-matched, population-based control individuals (median age: 68 years). Genetic investigation was carried out following both a coding SNP and haplotype tagging approach. Subgroup analyses for N = 143 patients with early manifestation of CRC (≤50 age at diagnosis) were performed for all *CARD *loci and subgroup analyses for diverse age strata were carried out for *CARD15 *mutations R702W, G908R and L1007fs. In addition, all SNPs were tested for association with disease presentation and family history of CRC.

**Results:**

No significant differences were observed between the patient and control allelic or haplotypic spectra of the three genes under study for the total cohort (N = 1044 patients). None of the analysed SNPs was significantly associated with either tumour location or yielded significant association in the familial or non-familial CRC patient subgroups. However, in a patient subgroup (≤45 age at diagnosis) with early disease manifestation the mutant allele of *CARD15 *R702W was found to be significantly associated with disease susceptibility (9.7% in cases *vs *4.6% in controls; P_allelic _= 0.008, P_genotypic _= 0.0008, OR_allelic _= 2.22 (1.21-4.05) OR_ressessive _= 21.9 (1.96-245.4).

**Conclusion:**

Variation in the innate immunity genes *CARD4*, *CARD8 *and *CARD15 *is unlikely to play a major role in the susceptibility to CRC in the German population. But, we report a significant disease contribution of *CARD15 *for CRC patients with very early disease manifestation, mainly driven by variant R702W.

## Background

Colorectal cancer (CRC) occurs both as a part of recognized heritable syndromes and in the form of "sporadic" disease. However, epidemiological studies have also revealed familial clustering of CRC outside the recognized syndromes [1]. Estimates of the relative familial recurrence risk of nonsyndromic CRC range from 1.7 for unselected cases [2] to 6.2 for siblings of index patients aged <55 years [3]. As yet, the molecular basis of this familial clustering of "sporadic" CRC has not been fully explored.

Epidemiological and functional evidence suggest that cancer may arise in the context of chronic inflammation. Inflammatory bowel disease (IBD) is a well established example in that patients with IBD have an increased risk for the development of CRC [4]. It is estimated that 1-2% of all CRC cases in general population are due to a complicated course of ulcerative colitis (UC) or Crohn disease (CD), and up to 15% of all patients with IBD develop CRC during their life [5,6]. Therefore, it appears sensible to hypothesize that genetic factors predisposing to, or involved in, the chronic inflammatory response in IBD also play an important role for the predisposition to CRC.

As regards CD, the *CARD15 *(caspase recruitment domain family, member 15) gene mutations rs2066844 (R702W), rs2066845 (G908R) and rs2066847 (L1007fs) were originally shown to be associated with an increase disease risk [7,8], and this association has since been replicated in numerous Caucasian populations [9-11]. The *CARD15 *gene overlaps with the linkage-based IBD1 locus on chromosome 16q12. It exerts its biological function as part of a larger molecular network of genes involved in innate immune recognition and regulation [12]. There are two other genes in this CARD family that have been implicated in the etiology of IBD as well. These include CARD4/NOD1 [13] and CARD8/TUCAN [14], both of which also function as intracellular receptors of bacterial products, but with different ligand specificity.

*CARD4/NOD1 *is located within a susceptibility locus for IBD on chromosome 7p14. CARD4 and is an additional PAMP receptor, that is linked to up apoptosis and activates NF-κB. The complex intronic indel polymorphism ND1+32656 (partially defined by rs6958571) has recently been reported to be associated with IBD [13]. *CARD8/TUCAN *(tumor-up-regulated CARD-containing antagonist of caspase nine) is expressed in gastrointestinal epithelium. There is evidence to suggest that CARD8 may be a negative regulator of NF-κB and also has a regulatory effect on apoptosis. The common variant rs2043211(c.30T>A) introduces a stop codon (Cys10Ter) at position 10 of the amino acid sequence. The common allele T was recently found to be associated with Crohn Disease [14].

Recently, several studies implicated *CARD15 *in the susceptibility to CRC. Thus, Kurzawski et al. [15] observed an increased frequency in comparison to newborns of the 3020InsC mutation among 250 Polish CRC patients aged >50 years at the time of diagnosis resulting in an odds ratio of 2.2. Interestingly, no evidence for an association with CRC was found in younger patients. The 3020InsC association was confirmed in a subsequent study from Greece [16] and for *CARD15 *R702W in a study from New Zealand [17]. However, three subsequent studies from Finnland [18,19] and Hungary [20] failed to corroborate any of these findings. A summary of *CARD15 *allele frequencies observed in different CRC populations is given in Table 1.

**Table 1:** Summary of association analyses and overview of allele frequencies of CARD15 mutations stratified by age and population.

--------------------------Current study---------------------------													
	Country, **Ref**.	Poland, [15]	Poland, [15]	Finnland, [18]	Germany	Germany	Germany	Germany	Germany	Greece, [16]	New Zealand, [17]	Hungary, [20]	Finnland, [18]
	Age at diagnosis	>50	≤ 50	≤ 50	≤ 45	≤ 50	≤ 60	>50	total	total	total	total	total
	N CRC	250	50	68	72	143	597	901	1044	104	133	194	953(926*)
	N controls	300	300	348	724	724	724	724	724	100	201	200	348
*R*702*W*													
CRC	% Allele freq.	/	/	/	9.7	7.7	5.4	4.7	5.1	4.8	7.1	1.8	2.2
Controls	% Allele freq.	/	/	/	4.6	4.6	4.6	4.6	4.6	1	3	1.5	2.1
	p value	/	/	/	**0.008**	**0.03**	0.30	0.83	0.5	0.02	0.03	0.78	0.88
	OR_Allelic_	/	/	/	2.22	1.75	1.2	1.04	1.13	5.21	2.3	1.21	1.04
	[95% CI]				[1.21--4.05]	[1.05--2.91]	[0.85--1.71]	[0.74--1.44]	[0.82--1.55]	[1.11--24.23]	[1.1--5]	[0.39--3.67]	[0.61 -- 1.78]
*G*908*R*													
CRC	% Allele freq.	/	/	/	2.1	2.1	1.4	1.4	1.5	8.65	2.2	1.8	0.3
Controls	% Allele freq.	/	/	/	1.2	1.2	1.2	1.2	1.2	3.5	0.8	1.8	0.2
	p value	/	/	/	0.35	0.36	0.57	0.59	0.43	0.025	0.09	0.95	0.57
	OR_Allelic_	/	/	/	1.79	1.69	1.22	1.18	1.27	2.78	3.1	1.03	1.59
	[95% CI]				[0.52--6.19]	[0.66--4.36]	[0.62--2.39]	[0.64--2.20]	[0.70--2.30]	[1.11--6.98]	[7.7--12.7]	[0.35--3.00]	[0.32 -- 7.91]
*3020InsC*													
CRC	% Allele freq.	14.4**	2**	2.2	5.6	3.8	3.9	3.6	3.6	12.5	2.2	3.6	1.9
Controls	% Allele freq.	7**	7**	1.9	2.8	2.8	2.8	2.8	2.8	6	1	2.5	1.9
	p value	0.0046	0.3	0.78	0.06	0.32	0.09	0.21	0.17	0.017	0.19	0.4	0.96
	OR_Allelic_	2.23	0.27	0.78	2.07	1.42	1.44	1.29	1.31	2.44	2.3	1.48	0.98
	[95% CI]	[1.23--4.10]	[0.01--1.97]	[0.17--3.54]	[0.95--4.51]	[0.71--2.83]	[0.94--2.21]	[0.87--1.93]	[0.89--1.93]	[1.15--5.17]	[0.64--8.4]	[0.64--3.41]	[0.51 -- 1.88]

* the initial investigation of 3020InsC utilised only 926 CRC cases ** genotype frequencies of carriership of mutant allele

In view of these controversial results, and in order to obtain a more complete assessment of the impact of variation in the innate immunity genes on CRC susceptibility, we investigated multiple variants of the *CARD4*, *CARD8 *and *CARD15 *genes in a large case-control sample from Northern Germany, following both a haplotype tagging and a coding SNP approach.

## Methods

### Patients and phenotypes

Patients with histologically confirmed colorectal carcinoma (CRC) were identified through the regional cancer registry of Schleswig-Holstein or through the records of surgical departments in Northern Germany. Patients logged in the registry or patients operated with sporadic CRC between 2002 and 2005 at hospitals in Kiel, Eckernförde, Rendsburg, Schleswig, Flensburg, Husum, Heide, Niebüll, Neumünster, Itzehoe, Rotenburg, Stade, Reinbek, Bad Oldesloh, Detmold, Neustadt, Hamburg-Harburg, Hamburg-Altona, Hamburg-Eilbek, Hamburg-Bergedorf, Hamburg-Barmbek, Bremen and Lüneburg were contacted by mail and invited for participation in this study. Patients who did not respond were sent one written reminder. Individuals who agreed to participate were contacted through the recruitment office of the POPGEN biobank http://www.popgen.de[21]. They were interviewed by mail questionnaire and a venous EDTA blood sample was obtained either at the POPGEN office or by the patient's general practitioner. All study protocols were approved by the institutional ethics committees and the local data protection officer. Written informed consent was obtained from all study participants. For both cases and controls, the study was restricted to probands of German ancestry, i. e. only individuals whose parents were born in Germany. Patients and controls fulfilling either of the clinical Amsterdam or Bethesda criteria for HNPCC were excluded for the study [22], as were patients with a history of inflammatory bowel disease (IBD). The first consecutive 522 male and 522 female participants were included in the study, yielding a sample size of 1044 patients. The median age at diagnosis was 59 years (range: 18-92 years; Table 2). Patients with FAP (as were all other known monogenic forms of CRC) were excluded from the study. Patients having at least 1 first-degree relative diagnosed with CRC were defined as familial cases, whereas non-familial cases had no first-degree relatives with CRC. Healthy control individuals (N = 724, including 362 males) with a median age at time of recruitment of 68 years (range: 48-81 years) were obtained from the population-derived pool of controls individuals in the POPGEN project, identified on the basis of the local population registry [21]. Control individuals with a history of malignant disease or IBD were excluded from the study. Controls were sex- and age-matched to the case sample, with a median age of 10 (± 1) years above the cases' age at diagnosis.

**Table 2:** Overview and clinicopathologic characteristics of the cohorts and subgroups used in the present study.

Sample	N	Median age	Median age at diagnosis	%male
Cases (total cohort)	1044	63	59	50%
Tumour location				
Colon (Subgroup 1)	539	63	59	47%
Rectum (Subgroup 2)	469	63	59	53%
Rectum and remaining colon	36	64	59	50%
Family history				
Familial CRC (Subgroup 3)	189	64	60	45%
Non-Familial CRC (Subgroup 4)	855	63	59	51%
Cases (age at diagnosis ≤ 60)	337	60	56	50%
Cases (age at diagnosis ≤ 50)	143	49	45	43%
Cases (age at diagnosis ≤ 45)	72	45	41	42%
Cases (age at diagnosis > 50)	901	64	60	50%
Controls (sexmatched to the total cohort)	724	68	/	50%
Controls (sexmatched to case ≤ 50)	639	68	/	43%

Subcohorts 1-4 were not significantly different in respect to tumor location and family history to the total CRC patient cohort.

### Genotyping

DNA from all samples was prepared using the FlexiGene chemistry (Qiagen, Hilden, Germany) according to the manufactures protocols. DNA samples were evaluated by gel electrophoresis and adjusted to 20-30 ng/μl DNA content using the Picogreen fluorescent dye (Molecular Probes - Invitrogen, Carlsbad, Ca, USA). One microliter of genomic DNA was amplified with the GenomiPhi (Amersham, Uppsala, Sweden) whole genome amplication kit and fragmented at 99°C for three minutes. One hundred nanograms of DNA were dryed overnight in TwinTec hardshell 384 well plates (Eppendorf, Hamburg, Germany) at room temperature. Genotyping was performed for these plates using the SNPlex chemistry (Applied Biosystems, Foster City, USA) on an automated platform with TECAN Freedom EVO and 384 well TEMO liquid handling robots (TECAN, Männedorf, Switzerland). Genotypes were reviewed manually using the Genemapper 4.0 (Applied Biosystems) software. None of the variants showed a significant departure from Hardy-Weinberg equilibrium (p > 0.1), indicating robust genotyping in this experiment. All process data were logged and administered with a database-driven LIMS [23].

Genotypes of nonsynonymous polymorphism R702W (rs2066844) and the complex intronic indel polymorphism ND1+32656 (PCR forward primer GTCCTTCTGGTGTACTGATGT ATGAAA, PCR reverse primer, Taqman probe VIC (T-allele): CGCCCCCCACACA, Taqman probe FAM [GG-allele): CCCCCCCCCACAC) were determined using the TaqMan (Applied Biosystems) system. Reactions were completed and read in a 7900 HT TaqMan sequence detector system (Applied Biosystems). The amplification reaction was carried out with the TaqMan universal master mix. Thermal cycling conditions consisted of 1 cycle for 10 minutes at 95°C, 45 cycles for 15 seconds at 95°C, and 45 cycles for 1 minute at 60°C. Primers and probes have been reported before [24].

### SNP selection and data analysis

**Figure 1:**
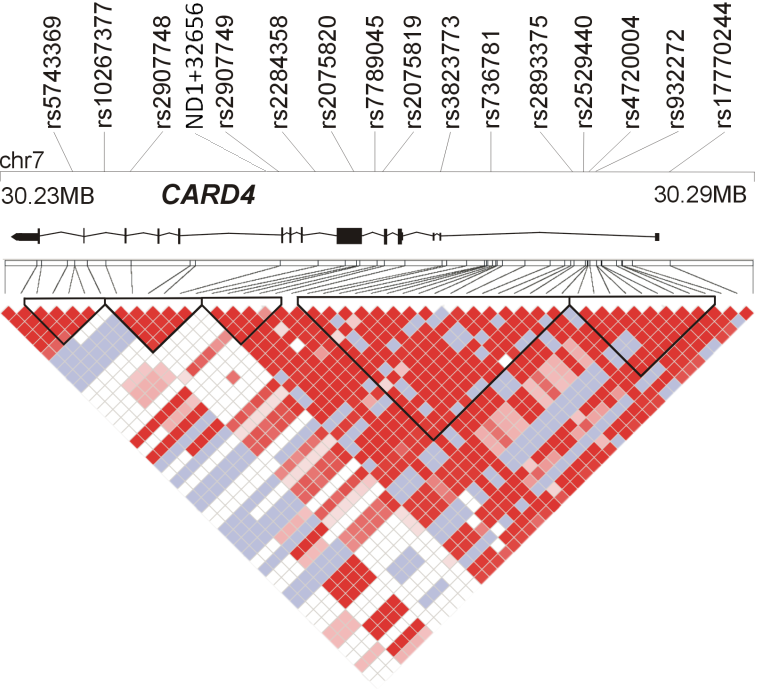
**Overview of the physical and genetic structure of the CARD4 gene region**. The gene is annotated on the genomic minus strand. The physical position of the investigated SNPs and a schematic illustration of the gene structure are shown in the top panel. The coordinates refer to genome assembly build 35. The lower panel gives an overview of the linkage disequilibrium structure of the locus [D'] as generated by Haploview [29]. The LD plots have been generated from the HAPMAP data.

**Figure 2:**
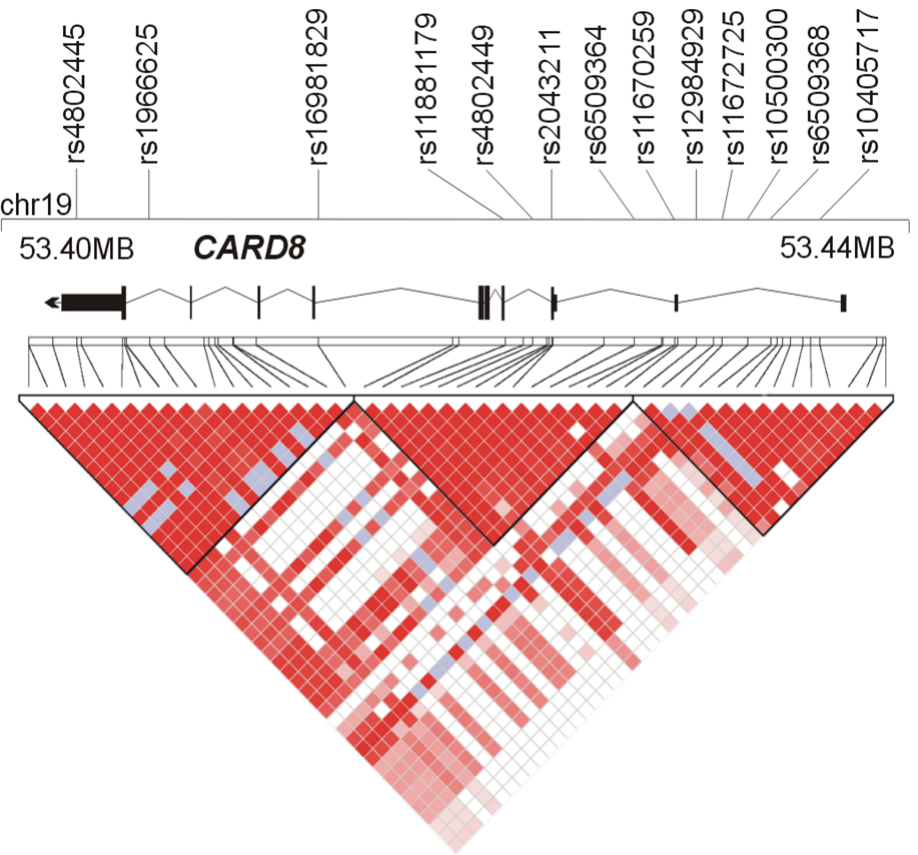
**Overview of the physical and genetic structure of the gene CARD8 gene region**. The gene is annotated on the genomic minus strand. For details, see legend to Figure 1.

**Figure 3:**
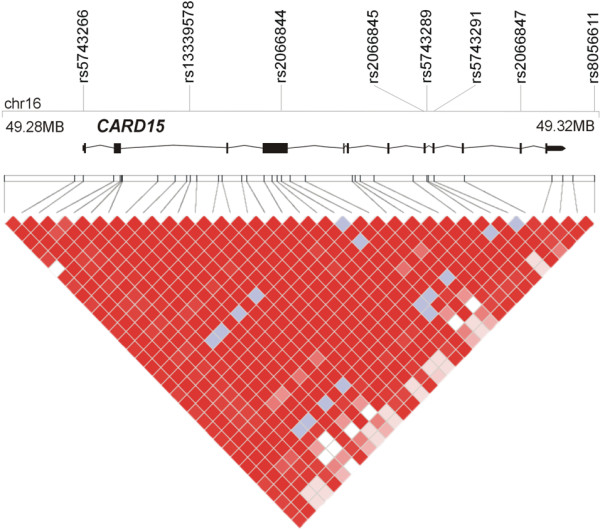
**Overview of the physical and genetic structure of the gene CARD15 gene region**: The gene is annotated on the genomic plus strand. For details, see legend to Figure 1.

For all genes, single nucleotide polymorphism (SNPs) were retrieved from HAPMAP http://www.hapmap.org by the automated selection, from the CEU dataset, of haplotype tagging SNPs for Causcasians (setting: Mendel errors: 0, minor allele frequency >0.05, HWE cut-off p > 0.01) [25]. In addition, coding SNPs reported in dbSNP or in the literature were included if they had a a minor allel frequency ≥0.01 in Caucasians. Figures 1, 2 and 3 show the distribution of markers across genes and the regional haplotype structures as generated by HAPLOVIEW.

The study was of case-control design. In order to improve power - i. e. to detect association to variants on the haplotypes, that are not directly tagged by one of the SNPs in the experiments - a sliding window haplotype analysis using window sizes of two to five markers was performed. Haplotype analysis was performed using COCAPHASE through the UNPHASED suite of programs http://www.rfcgr.mrc.ac.uk/~fdudbrid/software/unphased/[26]. COCAPHASE performs likelihood ratio tests under a log-linear model of the probability that a haplotype belongs to the case rather than to the control group. The expectation maximization (EM) algorithm is utilized to resolve uncertain haplotypes and provides maximum-likelihood estimates of frequencies. One single overall test statistic per sliding window (HAP2-5) is reported as a global significance value *P *for each haplotype tested. Nominal p values will be reported fo all tests. Single-point genotype- and allele-based tests of association were performed using a chi-squared test or fisher exact test.

## Results

**Figure 4:**
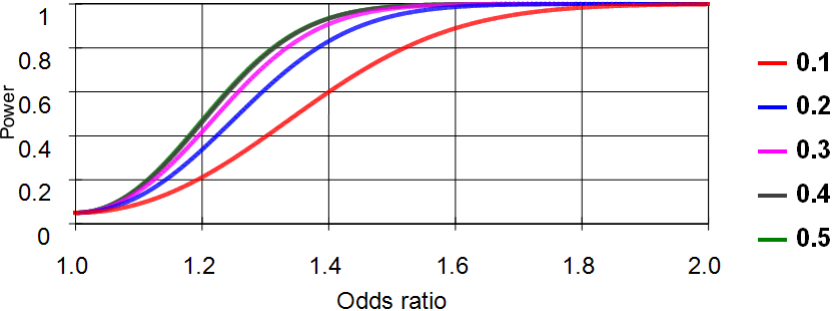
**Power analysis of the sample used in the present study**. The power of an allelic test is plotted as a function of the underlying odds ration of the tested genetic variant. Calculations were performed for a nominal significance level of 0.05 in a two-sided test. The different colors denote the frequency of the minor allele of the respective variant. Clearly, power increased for frequent variants and higher underlying odds. It is evident, that odds ratios above 1.6 should be detectable with a power greater 80% for all allele frequencies. The graph was generated using PS-power [27] and shows the power as a function of the odds ratio (x-axis).

A systematic power analysis was performed for the 1044 cases and 724 controls available for study, adopting various allelic odds ratio between 1 and 2, a nominal significance level of 0.05, and minor allele frequencies of 0.1, 0.2, 0.3, 0.4 and 0.5 for a potential susceptibility mutation, respectively [27]. The power to detect odds ratios >1.5 was found to be >80% under all models (Figure 4). For frequent susceptibility factors, even odds ratios >1.3 would be detectable with the same power.

All tagging SNPs (see Methods section) from the three candidate genes and all validated nonsynonymous SNPs with a minor allele frequency ≥1% in Caucasians were included in the genotyping. Sixteen SNPs in the *CARD4 *gene, 13 SNPs in the *CARD8*, and 8 SNPs in the *CARD15 *gene were selected. These markers provided a good coverage of the respective genes and tagged all major haplotype blocks as determined by the tagging routine [28] implemented in HAPLOVIEW [29]. The disease association analyses were performed in single-tier fashion, using all cases and controls. Because earlier reports have indicated age-stratification of *CARD15 *association and younger patients in general may have a stronger genetic component to their disease, a separate analysis including only cases with an age of onset below 50 (median age of onset 45) compared to a sexmatched control population was performed, too. In addition, a further subgroup analysis for cases with an age of onset below 45 (median age of onset 41) was performed for *CARD15 *risk variants, too.

### CARD4

For *CARD4*, some 16 SNP markers were genotyped. Figure 1 provides an overview of the linkage disequilibrium pattern generated from Hapmap Caucasian samples and the location of the tag SNPs in the gene. Association findings are summarised in Table 3. Nominal p-values for allelic association tests in the total cohort ranged from 0.10 to 0.92; sliding window haplotype analyses of two to five markers yielded p values between 0.08 and 0.99, respectively. The complex intronic indel polymorphism ND1+32656 yielded a p-value of 0.13 for the total cohort. ND1+32656 is part of a conserved haplotype also defined by SNPs rs2907748 and rs2907749 [30]. Both SNPs rs2907748 and rs2907749 (31 bp downstream to rs6958571) were also included in our experiments for ease and robustness of genotyping [30] and yielded similar p-values as ND1+32656. The non-synonymous coding SNP E266K, which was reported to be associated with disease susceptibility for Crohn's disease [31] in Hungarian patients, yielded a p-value of 0.36 for the total cohort. In the analysis of the total patient sample, none of the single point or haplotype analyses revealed any significant association with CRC risk.

**Table 3:** Results of the association analyses of tagging SNPs in the *CARD4 *gene.

dbSNP id	*CARD4* Position	MAF case	MAF cont	**OR**_**allelic**_**[CI_**95%**_]**	**P**_**allelic**_	**P**_**HAP2**_	**P**_**HAP3**_	**P**_**HAP4**_	**P**_**HAP5**_
***1044 CRC patients vs 724 sex-matched control individuals***									
Rs5743369	intron	0.15	0.16	0.93 [0.78-1.12]	0.445	0.371	0.252	0.333	0.421
Rs10267377	intron	0.30	0.28	1.09 [0.94-1.27]	0.253	0.222	0.322	0.556	0.274
Rs2907748	intron	0.26	0.23	1.14 [0.98-1.34]	0.103	0.415	0.748	0.637	0.546
ND1+32656	intron	0.26	0.24	1.13 [0.96-1.32]	0.131	0.512	0.405	0.238	0.148
Rs2907749	intron	0.29	0.27	1.1 [0.95-1.28]	0.234	0.365	0.095	0.112	0.199
Rs2284358	intron	0.29	0.27	1.09 [0.94-1.26]	0.304	0.618	0.152	0.204	0.184
Rs2075820	*E*266*K*	0.25	0.27	0.93 [0.80-1.09]	0.363	0.666	0.367	0.374	0.294
Rs7789045	intron	0.46	0.47	0.96 [0.84-1.10]	0.558	0.755	0.366	0.35	0.384
rs2075819	intron	0.27	0.29	0.92 [0.79-1.07]	0.307	0.092	0.086	0.22	0.343
rs3823773	intron	0.12	0.12	0.98 [0.79-1.21]	0.891	0.984	0.986	0.864	0.382
rs736781	intron	0.19	0.19	0.99 [0.83-1.17]	0.919	0.941	0.765	0.283	0.352
rs2893375	intron	0.17	0.17	1.03 [0.86-1.23]	0.783	0.911	0.162	0.255	0.172
rs2529440	intron	0.42	0.42	1.02 [0.89-1.17]	0.777	0.364	0.243	0.175	-#
rs4720004	intron	0.15	0.14	1.13 [0.93-1.37]	0.236	0.525	0.327	-#	-#
rs932272	intron	0.40	0.39	1.04 [0.91-1.20]	0.536	0.286	-#	-#	-#
rs17770244	intron	0.06	0.06	1.01 [0.76-1.35]	0.927	-#	-#	-#	-#
***143 CRC patients (age at diagnosis ≤ 50) vs 639 sex-matched control individuals***									
rs5743369	intron	0.12	0.16	0.7 [0.47-1.03]	0.065	0.069	0.09	0.19	0.22
rs10267377	intron	0.34	0.29	1.26 [0.96-1.66]	0.093	0.212	0.41	0.55	0.36
rs2907748	intron	0.28	0.23	1.3 [0.97-1.74]	0.075	0.273	0.52	0.32	0.15
ND1+32656	intron	0.29	0.24	1.31 [0.98-1.76]	0.065	0.263	0.13	0.04	0.10
rs2907749	intron	0.32	0.27	1.25 [0.95-1.66]	0.111	0.06	0.007	0.04	0.10
rs2284358	intron	0.32	0.28	1.25 [0.95-1.65]	0.117	0.01	0.02	0.06	0.07
rs2075820	*E*266*K*	0.19	0.27	0.62 [0.45-0.86]	0.004^a^	0.009	0.03	0.04	0.06
rs7789045	intron	0.43	0.47	0.84 [0.65-1.09]	0.188	0.01	0.01	0.04	0.06
rs2075819	intron	0.21	0.29	0.62 [0.46-0.85]	0.003^a^	0.005	0.01	0.02	0.04
rs3823773	intron	0.09	0.12	0.69 [0.44-1.07]	0.098	0.07	0.08	0.11	0.03
rs736781	intron	0.14	0.2	0.66 [0.46-0.95]	0.025^a^	0.03	0.05	0.01	0.02
rs2893375	intron	0.21	0.17	1.3 [0.94-1.79]	0.110	0.07	0.01	0.03	0.02
rs2529440	intron	0.41	0.42	0.96 [0.74-1.24]	0.750	0.01	0.03	0.02	-#
rs4720004	intron	0.19	0.14	1.5 [1.07-2.09]	0.017^a^	0.03	0.03	-#	-#
rs932272	intron	0.39	0.39	0.98 [0.75-1.28]	0.887	0.76	-#	-#	-#
rs17770244	intron	0.06	0.06	1.11 [0.65-1.89]	0.704	-#	-#	-#	-#

^a ^P value were not significant after Bonferroni correction for multiple testing, *no odds ratio calculated owing to low allele frequency, ^# ^p value reported for the first marker in the haplotype. The minor allele frequencies (MAF) for cases and controls are reported. P values and odds ratios are reported for the allelic (p_allelic_) test. Columns P_HAP2 _to P_HAP5 _refer to a sliding window haplotype analysis using COCAPHASE. For example, P_HAP3 _(0.252) for rs5743369 reports the global significance value for the window 3 haplotype spanning rs5743369- rs10267377- rs2907748.

The subgroup analysis of patients younger than 50 years at onset of disease for ND1+32656 yielded an allelic p-value of 0.06 (Table 2). The subgroup analysis of the most youngest CRC patients (N = 72; ≤45 age at diagnosis) yielded (P_allelic _= 0.06, P_genotypic _= 0.025, OR_allelic _= 1.29 (0.79-2.11) and did not improve the results. For E266K a significant association was observed in patients younger than 50 years (P_allelic _= 0.004, OR_allelic _= 0.62 (0.45-0.86). A similarly p-value of 0.003 was yielded for the intronic tag SNP rs2075819 that is in strong LD (r^2 ^= 0.9) to E266K. The subgroup analysis of the most youngest CRC patients (<45) for E266K yielded (P_allelic _= 0.03, P_genotypic _= 0.014, OR_allelic _= 0.48 (0.28-0.82)). However, both E266K and rs2075819 only have borderline significance in respect to a significance threshold of p 0.003 after Bonferroni correction for multiple testing (N = 16 SNP marker).

### CARD8

In total, 13 SNPs in the *CARD8 *gene were genotyped (Figure 2). In our study, neither allele of rs2043211 was associated with CRC (P_allelic _= 0.86 OR_allelic _= 1.01 (0.88-1.17). Variant rs11881179 (c.202A>G) is a coding SNP that leads to the substitution of valin by isoleucin at position 68 of the amino acid sequence. No difference in terms of the minor allele frequency (MAF) was observed between cases and controls (MAF:c.202G_cases _= 0.1%, c.202G_controls _= 0.1%, P_allelic _= 0.965). The Caucasians allele frequencies in HAPMAP and the observed allele counts in our control group (Table 4) were very similar (p > 0.2), thereby justifying the use of HAPMAP for the selection of tagging SNPs. All remaining single point allelic and genotypic association tests resulted in nominal p-values between 0.09 and 0.99. Haplotype analysis yielded the lowest p value of 0.23 for a 3-locus haplotype spanning from rs10500300 to rs10405717.

**Table 4:** Results of the genetic association analyses of the tagging SNPs in the *CARD8 *gene.

dbSNP id	*CARD8* Position	MAF case	MAF cont	**OR**_**allelic**_**[CI_**95%**_]**	**P**_**allelic**_	**P**_**HAP2**_	**P**_**HAP3**_	**P**_**HAP4**_	**P**_**HAP5**_
***1044 CRC patients vs 724 sex-matched control individuals***									
rs4802445	3'UTR	0.44	0.45	0.95 [0.83-1.08]	0.431	0.549	0.540	0.615	0.305
rs1966625	intron	0.09	0.10	0.90 [0.72-1.13]	0.360	0.552	0.463	0.368	0.726
rs16981829	intron	0.33	0.33	1.00 [0.86-1.15]	0.976	0.998	0.419	0.712	0.697
rs11881179	*V*68*I*	0.001	0.001	1.04 [0.17-6.25]	0.964	0.975	0.999	0.971	0.518
rs4802449	intron	0.34	0.34	0.99 [0.86-1.14]	0.856	0.966	0.828	0.406	0.488
rs2043211	*C10Ter*	0.32	0.32	1.01 [0.88-1.17]	0.857	0.907	0.474	0.477	0.549
rs6509364	intron	0.34	0.34	1.02 [0.88-1.17]	0.820	0.593	0.642	0.661	0.602
rs11670259	intron	0.35	0.34	1.05 [0.91-1.21]	0.486	0.483	0.592	0.675	0.517
rs12984929	intron	0.41	0.39	1.09 [0.95-1.25]	0.235	0.545	0.760	0.343	0.417
rs11672725	intron	0.20	0.19	1.02 [0.86-1.21]	0.819	0.819	0.865	0.350	-#
rs10500300	intron	0.08	0.08	1.06 [0.83-1.36]	0.619	0.820	0.226	-#	-#
rs6509368	intron	0.44	0.44	1.02 [0.89-1.16]	0.816	0.544	-#	-#	-#
rs10405717	intron	0.15	0.17	0.90 [0.75-1.07]	0.238	-#	-#	-#	-#
***143 CRC patients (age at diagnosis ≤50) vs 639 sex-matched control individuals***									
rs4802445	3'UTR	0.4	0.45	0.81 [0.62-1.05]	0.106	0.197	0.310	0.352	0.321
rs1966625	intron	0.07	0.1	0.73 [0.45-1.18]	0.200	0.322	0.373	0.388	0.692
rs16981829	intron	0.31	0.33	0.94 [0.71-1.24]	0.663	0.603	0.388	0.647	0.729
rs11881179	*V*68*I*	0	0.002	N/A*	0.503	0.166	0.280	0.372	0.039
rs4802449	intron	0.39	0.34	1.25 [0.96-1.63]	0.100	0.232	0.341	0.028	0.131
rs2043211	*C10Ter*	0.29	0.32	0.84 [0.63-1.11]	0.225	0.251	0.024	0.144	0.067
rs6509364	intron	0.32	0.33	0.94 [0.72-1.24]	0.668	0.004	0.031	0.020	0.092
rs11670259	intron	0.43	0.34	1.46 [1.13-1.90]	**0.004**^a^	0.023	0.018	0.118	0.159
rs12984929	intron	0.37	0.39	0.93 [0.71-1.21]	0.595	0.865	0.441	0.332	0.407
rs11672725	intron	0.2	0.2	1.04 [0.75-1.43]	0.830	0.755	0.575	0.615	-#
rs10500300	intron	0.09	0.08	1.14 [0.72-1.80]	0.583	0.590	0.750	-#	-#
rs6509368	intron	0.47	0.44	1.1 [0.85-1.42]	0.479	0.585	-#	-#	-#
rs10405717	intron	0.17	0.16	1.08 [0.77-1.52]	0.648	-#	-#	-#	-#

^a ^P value were not significant after Bonferroni correction for multiple testing, *no odds ratio calculated due to low allele frequency, ^# ^p-value is reported for the first marker in the haplotype window. The minor allele frequencies (MAF) for cases and controls are reported. P values and odds ratios are reported for the allelic (p_allelic_) test. Columns P_HAP2 _to P_HAP5 _refer to a sliding window haplotype analysis using COCAPHASE. For example, P_HAP5 _(0.417) for rs12984929 reports the global significance value for the window 5 haplotype spanning rs12984929-rs11672725- rs10500300- rs6509368- rs10405717.

The subgroup analysis of younger patients (≤50 age at diagnosis) for rs2043211 yielded a similar allelic p-value of 0.23 (Table 4). The intronic SNP rs11670259 was significantly associated with CRC (P_allelic _= 0.004, OR_allelic _= 1.46 (1.13-1.90), but remains only borderline significant in respect to a significance threshold of p 0.004 after Bonferroni correction for multiple testing (N = 13 SNP marker). None of the other single marker or haplotyp SNPs at that locus yielded significant p values after Bonferroni correction was applied.

### CARD15

Eight SNP markers in the *CARD15 *gene were genotyped (Figure 3) and the results of the respective association analyses are summarised in Table 1, 5, 6 &7. The haplotype structure and the risk alleles at *CARD15 *are well defined [32], so that the initial analyses focussed on the three main coding SNPs (Table 5). No association was seen between CRC and *CARD15 *variants R702W (5.1% in cases *vs *4.6% in controls; P_allelic _= 0.50, P_genotypic _= 0.59), G908R (1.5% *vs *1.2%; P_allelic _= 0.43, P_genotypic _= 0.60) and L1007fs (3.6% *vs *2.8%; P_allelic _= 0.17, P_genotypic _= 0.36). Two compound heterozygotes carrying R702W and G908R and four compound heterozygotes carrying R702W and L1007fs were observed among the CRC patients, whereas non of these combinations was found in controls. The combined frequency of genotypes harbouring R702W, G908R or L1007fs was also not significantly different in cases and controls (10.2% *vs *8.6%; P_allelic _= 0.10, P_genotypic _= 0.10).

**Table 5:** Results of the genetic association analyses of the tagging SNPs in the *CARD15 *gene.

dbSNP id	*CARD15 *Position	MAF case	MAF cont	**OR**_**allelic**_**[CI_**95%**_]**	**P**_**allelic**_	**P**_**HAP2**_	**P**_**HAP3**_	**P**_**HAP4**_	**P**_**HAP5**_
***1044 CRC patients vs 724 sex-matched control individuals***									
rs5743266	5'UTR	0.30	0.29	1.05 [0.91-1.22]	0.459	0.687	0.236	0.362	0.517
rs13339578	intron	0.30	0.30	0.99 [0.86-1.15]	0.980	0.835	0.903	0.443	0.209
rs2066844	*R*702*W*	0.05	0.05	1.13 [0.82-1.55]	0.501	0.838	0.391	0.323	0.317
rs2066845	*G*908*R*	0.01	0.01	1.27 [0.70-2.30]	0.432	0.353	0.269	0.266	0.169
rs5743289	intron	0.18	0.17	1.11 [0.93-1.33]	0.247	0.103	0.151	0.132	-#
rs5743291	*I*955*V*	0.08	0.10	0.80 [0.64-1.01]	0.052	0.041	0.121	-#	-#
rs2066847	*L1007fs*	0.04	0.03	1.31 [0.89-1.93]	0.171	0.295	-#	-#	-#
rs8056611	3'flank. region	0.49	0.49	1.02 [0.89-1.17]	0.823	-#	-#	-#	-#
***143 CRC patients (age at diagnosis ≤50) vs 639 sex-matched control individuals***									
rs5743266	5'UTR	0.31	0.29	1.11 [0.84-1.47]	0.456	0.232	0.196	0.232	0.323
rs13339578	intron	0.32	0.3	1.09 [0.83-1.44]	0.521	0.070	0.145	0.106	0.153
rs2066844	*R*702*W*	0.08	0.05	1.75 [1.05-2.91]	**0.029**^a^	0.03	0.117	0.210	0.228
rs2066845	*G*908*R*	0.02	0.01	1.69 [0.66-4.36]	0.273	0.161	0.305	0.275	0.281
rs5743289	intron	0.21	0.17	1.29 [0.94-1.78]	0.118	0.323	0.185	0.183	-#
rs5743291	*I*955*V*	0.08	0.1	0.74 [0.46-1.19]	0.214	0.116	0.598	-#	-#
rs2066847	*L1007fs*	0.04	0.03	1.42 [0.71-2.83]	0.316	0.613	-#	-#	-#
rs8056611	3'flank. region	0.5	0.48	1.05 [0.81-1.36]	0.692	-#	-#	-#	-#

^a ^P value were not significant after Bonferroni correction for multiple testing, *no odds ratio calculated due to low allele frequency, ^# ^p-value reported for the first marker in the haplotype window. The minor allele frequencies (MAF) for cases and controls are reported. P values and odds ratios are reported for the allelic (p_allelic_) test. Columns P_HAP2 _to P_HAP5 _refer to a sliding window haplotype analysis using COCAPHASE. For example, P_HAP2 _(0.295) for rs2066847 reports the global significance value for the window 2 haplotype sapnning rs2066847- rs8056611.

**Table 6:** Genotype and allele frequencies for *CARD15 *mutations among German CRC patients and controls.

*CARD15*		N	1*1	1*2	2*2	1*2 (c)	2*2 (c)	R702W-G908R	R702W - 1007fs	**P**_**allelic**_	**P**_**geno**_	**P**_**geno**_**(c)**^**a**^	**OR**_**CAR**_**(c)** **[CI_**95%**_]**^**a**^	**OR**_**REC**_**(c)** **[CI_**95%**_]**^**a**^
R702W	≤45CRC	72	60	10	2	9	3	0	1	0.008^b^	0.0008^b^	1.3 • 10^-5 b^	1.99 [1.02-3.89]^b^	32.9 [3.36-321.2]^b^

	≤50CRC	143	123	18	2	17	3	0	1	0.029	0.033	0.0062	1.66 [0.96-2.86]	14.2 [1.46-137.6]

	Control	639	582	56	1	56	1							

	CRC	1044	941	99	4	93	10	2	4	0.501	0.591	0.098	1.09 [0.78-1.51]	6.2 [0.79-48.4]

	Control	724	658	65	1	65	1							

G908R	≤45CRC	72	69	3	0	69	0			0.350^b^	n/a	n/a	1.81 [0.51-6.32]^b^	N/A

	≤50CRC	143	137	6	0	137	0			0.272	n/a	n/a	1.71 [0.65-4.43]	N/A

	Control	639	623	16	0	623	0							

	CRC	1044	1014	29	1	27	3	2	0	0.432	0.603	0.335	1.23 [067-2.24]	N/A

	Control	724	707	17	0	707	0							

L1007fs	≤45CRC	72	64	8	0	7	1	0	1	0.062^b^	0.086^b^	0.077^b^	2.25 [1.00-5.04]^b^	N/A

	≤50CRC	143	132	11	0	10	1	0	1	0.316	0.319	0.459	1.53 [0.75-3.01]	N/A

	Control	639	606	31	2	31	2							

	CRC	1044	972	69	3	65	7	0	4	0.171	0.358	0.272	1.33 [0.89-2.00]	1.1 [0.17-6.35]

	Control	724	686	36	2	686	2							

R702W/G908R/L1007fs	≤45CRC	72	50	20	2	19	3	0	1	0.001^b^	0.002^b^	0.0001^b^	2.19 [1.28-3.75]^b^	12.1 [2.37-61.31]^b^

	≤50CRC	143	107	34	2	33	3	0	1	0.013	0.038	0.015	1.69 [1.09-2.60]	4.9 [0.99-25.01]

	Control	639	533	103	3	103	3							

	CRC	1044	845	191	8	185	14	2	4	0.175	0.347	0.099	1.17 [0.91-1.50]	3.3 [0.95-11.63]

	Control	724	603	118	3	118	3							

1*1 homozygous wild-type, 1*2 heterozygous, 2*2 homozygous mutant, **(c) **homozygous for mutant allele and compound heterozygous combined; P_allelic _and P_geno _are calculated from the observed genotypes (1*1, 1*2, 2*2);^a ^P_geno _2*2 (c): genotypic p values and OR_CAR_(c): *odds ratio for carriership of rare allele *and OR_REC_(c): *odds ratio for homozygosity of rare allele under the ressesive disease model *were calculated by judging compound heterozygotes as homozygotes of the rare allele, P_allelic _and P_geno _were calculated without considering compound heterozygotes; ^b^P values and odds ratios for young CRC (≤45 age at diagnosis) were calculated against the total control cohort.

**Table 7:** Results of the genetic association analyses for subgroups stratified by disease presentation and family history of disease.

Subgroups	Gene/Variant	N CRC	N CON	minAFca	minAFco	P allelic	OR allelic
***CARD4***							
Colon (Subgroup 1)	ND1+32656	526	720	0.253	0.236	0.336	1.09 [0.91-1.32]
Rectum (Subgroup 2)	ND1+32656	459	720	0.265	0.236	0.117	1.16 [0.96-1.41]
Familial (Subgroup 3)	ND1+32656	187	720	0.283	0.236	0.058	1.28 [0.99-1.65]
Non-Familial (Subgroup 4)	ND1+32656	832	720	0.253	0.236	0.276	1.10 [0.93-1.29]
							
Colon (Subgroup 1)	E266K	535	709	0.252	0.267	0.423	0.93 [0.77-1.11]
Rectum (Subgroup 2)	E266K	469	709	0.252	0.267	0.418	0.92 [0.77-1.12]
Familial (Subgroup 3)	E266K	188	709	0.247	0.267	0.451	0.90 [0.70-1.18]
Non-Familial (Subgroup 4)	E266K	852	709	0.254	0.267	0.429	0.94 [0.80-1.10]
***CARD8***							
Colon (Subgroup 1)	C10Ter	468	722	0.312	0.321	0.632	1.05 [0.89-1.24]
Rectum (Subgroup 2)	C10Ter	537	722	0.332	0.321	0.558	0.96 [0.80-1.14]
Familial (Subgroup 3)	C10Ter	189	722	0.317	0.321	0.886	0.98 [0.77-1.25]
Non-Familial (Subgroup 4)	C10Ter	852	722	0.326	0.321	0.794	1.02 [0.88-1.19]
***CARD15***							
Colon (Subgroup 1)	R702W	539	723	0.048	0.046	0.760	1.06 [0.73-1.54]
Rectum (Subgroup 2)	R702W	469	723	0.055	0.046	0.283	1.23 [0.85-1.78]
Familial (Subgroup 3)	R702W	189	723	0.029	0.046	0.155	0.63 [0.33-1.20]
Non-Familial (Subgroup 4)	R702W	855	723	0.056	0.046	0.183	1.24 [0.90-1.72]
							
Colon (Subgroup 1)	G908R	539	723	0.017	0.012	0.293	1.43 [0.73-2.79]
Rectum (Subgroup 2)	G908R	469	723	0.014	0.012	0.650	1.18 [0.57-2.45]
Familial (Subgroup 3)	G908R	189	723	0.019	0.012	0.305	1.59 [0.65-3.86]
Sporadic (Subgroup 4)	G908R	855	723	0.014	0.012	0.570	1.20 [0.64-2.24]
							
Colon (Subgroup 1)	L1007fs	539	724	0.040	0.028	0.088	1.46 [0.94-2.26]
Rectum (Subgroup 2)	L1007fs	469	724	0.032	0.028	0.542	1.16 [0.72-1.88]
Familial (Subgroup 3)	L1007fs	189	724	0.042	0.028	0.141	1.55 [0.86-2.81]
Sporadic (Subgroup 4)	L1007fs	855	724	0.035	0.028	0.273	1.26 [0.84-1.89]

Although the haplotype structure of *CARD15 *has been explored before, we performed a sliding window haplotype analysis of this locus for the sake of consistency. The results reflected the pattern seen in the coding SNP analysis (Table 5) and were essentially negative: Only a nominal significance level of 0.04 was obtained for the 2-locus haplotype spanning rs5743291 and rs2066847. None of the neighbouring haplotypes showed any evidence for an association with CRC.

The analysis of the younger patient groups in contrast yielded a significant association of *CARD15 *R702W (rs2066844) with CRC (7.7% in cases *vs *4.6% in controls; P_allelic _= 0.029, P_genotypic _= 0.033, OR_allelic _= 1.75 (1.05-2.91), OR_ressessive _= 9.46 (0.85-105.2). The association was even more pronounced when the most youngest CRC patients (≤45 age at diagnosis) were analysed (9.7% in cases *vs *4.6% in controls; P_allelic _= 0.008, P_genotypic _= 0.0008, OR_allelic _= 2.22 (1.21-4.05), OR_ressessive _= 21.9 (1.96-245.4). The p value yielded for the genotypic association remains significant after Bonferroni correction. G908R and L1007fs were not found to be significantly associated with CRC in any of the analysed age groups.

The combined frequency of genotypes harbouring R702W, G908R or L1007fs were significantly higher in patient than controls for the youngest CRC patient group (≤45 age at diagnosis) (16.7% in cases *vs *8.6% in controls; P_allelic _= 0.0014, P_genotypic _= 0.0021, OR_ressessive _= 12.1 (2.37-61.36), both p-values remained significant after Bonferroni correction. When compound heterozygotes were classified as being equal to homozygotes of the mutant allele for R702W (P_genotypic _= 0.000013, OR_carriership _= 1.99 (1.02-3.89), OR_ressessive _= 32.9 (3.36-321.2) was yielded and for combined frequency of genotypes harbouring R702W, G908R or L1007fs R702W (P_genotypic _= 0.0001, OR_carriership _= 2.19 (1.28-3.75), OR_ressessive _= 12.1 (2.37-61.31), respectively (Table 6).

In contrast, R702W was not significant in the more older patient group (N = 901, >50 age at diagnosis), (4.7% in cases *vs *4.6% in controls; P_allelic _= 0.83) (Table 1). Similar negative results for R702W were obtained when patients diagnosed with CRC before age 60 (N = 597) were analysed (5.4% in cases *vs *4.6% in controls; P_allelic _= 0.3).

### Stratification analysis of disease presentation and family history

None of the analysed SNPs for *CARD4, 8 *and *15 *was significantly associated (all p values > 0.05; Table 7) with tumour location or yielded significant association in the familial or non-familial CRC patient subgroups (Table 7 only lists a small selection of the analysed SNPs).

## Discussion

The functional relationship between inflammation and cancer is well established and dates back to Virchow in 1863. He propagated this interesting hypothesis based on the observation that some classes of irritants, together with the tissue injury and causative inflammation, enhance cell proliferation. By now it is known that many cancers arise from sites of infection, chronic irritation and inflammation. About 15% of the global cancer burden is attributable to infectious agents, and inflammation is a major element of these chronic infections. For example, the development of mucosal associated lymphoid tissue (MALT) B cell lymphoma and gastric cancer is associated with *Helicobacter pylori*-induced chronic gastritis, and an increased risk of CRC accompanies inflammatory bowel disease (IBD) [4,33-35]. Therefore, we tried to replicate previously reported associations of functional SNPs in innate immunity gene *CARD15 *with CRC susceptibility, and also investigated variants of the *CARD4 *and *CARD8 *innate immune genes in this context.

The international HAPMAP project http://www.hapmap.org has generated a whealth of genotype and marker information that significantly facilitates the design of candidate gene studies [25]. For our candidate gene study, a primary haplotype tagging approach was chosen, i.e. the genetic variation at both loci was captured by a set of carefully selected SNPs. This tagging approach is able to detect signals from hitherto unknown regulatory or functional elements in a given genetic region [28,36,37]. Therefore, it offers potential advantages over a direct mutation screen of the coding region of a gene because disease susceptibility may also be conferred by variations, for instance, in splice sites or intronic enhancers [38,39]. Tagging SNPs were thus selected from the public HAPMAP http://www.hapmap.org resources. The genotype and allele frequencies for the SNPs investigated in our control population were not significantly different from those of the Caucasian HAPMAP individuals (Tables 3, 4 and 5), thereby justifying the selection of tag SNPs from this resource [25].

We utilized over 1000 patients who have been operated for CRC, which renders our study the largest case-control study of *CARD15 *mutations reported so far. In Germany, the population median age of affection by CRC is about 69 years for males and 74 years for females [40]. The actual median age of onset in our cases was 59 years. In an additional attempt to increase the power of our investigations, only IBD-negative and cancer-free control individuals with a moderately higher median age of 68 years were used. Many polygenic disorders are characterized by a strong correlation between the age of onset of relatives, as has been documented for instance in breast cancer [41] and Alzheimer disease [42]. It is indeed plausible that the genetic influence upon the development of these disorders is partly reflected by the age at which individuals develop the disorder [43]. Confounding by population affiliation was minimized by the restriction to patients of German ancestry as determined by the birth place of both parents.

The sample size used in this study has a power >80% for the detection of allelic odds ratios >1.5 at a significance level of 0.05 (Figure 4). For more frequent mutation variants, even odds ratios as small as 1.3 would have been detected with the same power.

This notwithstanding, none of the single point nominal p values in any of the tests in the total cohort (N = 1044 CRC patients) were found to be <0.05. Thus, in contrast to Kurzawski et al. (2004) [15], Papaconstantinou et al. (2005) [16] and Roberts et al. (2006) [17], we found no evidence for an association between *CARD15 *variants and CRC susceptibility based on the findings of the total cohort.

However, in patient subgroups of early disease manifestation (≤45 and ≤50 age at diagnosis) we detected a significant association of the R702W mutation with CRC. In the German cohort, this association would have remained undiscovered if the subgroup analyses for earlier disease manifestation would had been confined only to patients with ≤60 years at diagnosis, as done in previous studies that did not detect an association with CRC [18-20]. In line with this, the association signal for all tested *CARD15 *mutations totally fainted when CRC patients older than 50 years at diagnosis were analysed. No association was found for *G908R*. We observed an increase in the frequency of R702W and also 3020insC risk alleles and compound heterozygotes between both with decreasing age of diagnosis in non-IBD affected CRC patients. Thus, *CARD15 *mediated CRC disease susceptibility seems to be confined to early onset CRC in the German population. *CARD15 *R702W and 3020insC affect the C-terminal LRR-part of NOD2 lead to a reduced responsiveness to bacterial components and are assumed to influence the crosstalk with Toll-like receptor function that results in a proinflammatory cytokine bias [44-46]. The resulting chronic imflammatory state of the colon could provide the mechanistic link to cancer development.

In the whole, however, our findings corroborate the two studies from Finnland and Hungary. Both studies disproved *CARD15 *mutations as major contributors to general 'sporadic' colorectal cancer disease susceptibility. In addition, none of the analysed SNPs was significantly associated with either tumour location or yielded significant association in the familial or non-familial CRC patient subgroups.

Furthermore, our study did not unravel any major contribution of *CARD4 *and *CARD8 *variants to the predisposition to CRC despite the results of recent genome-wide association studies [47-54] showing that part of the CRC risk is due to common low-risk variants. The intronic complex indel polymorphism *CARD4 *ND1+ 32656 was not significantly disease associated neither in the total cohort nor in the subgroup analysis of younger CRC patients. The coding polymorphism *CARD4 *E266K was reported to be associated with Crohn's disease in Hungarian population with the risk allele being more frequent in patients than in controls [31]. In contrast, we observed the E266K mutant allele 8% less frequently in the younger subgroup of CRC cases (≤50 age at diagnosis) compared to controls. Interestingly, in a Scottish IBD population the mutant allele was likewise observed less frequently, though not significant, in early onset IBD (<17 age) patients compared to controls [55].

There may be many explanations of the lack of significant results for the total CRC cohort in the present study. A major reason might be population genetic differences in terms of allele frequencies and in terms of the contribution of individual risk variants. Regional heterogeneity within Europe as reported with respect to the contribution of *CARD15 *variants to CD susceptibility [32,55] may also apply to the CRC risk. In addition, environmental factors may differ widely even between Caucasian populations.

Another critical point may be the necessity of a sufficient sample size utilized in association studies. There might be a weakness in this promise of previously published data, as studies demonstrating evidence of *CARD15 *as a susceptibility gene for CRC, named Kurzawski *et al*. [15] and Papaconstantinou *et al*. [16], feature relatively low patient numbers.

## Conclusion

In conclusion, common variants of the innate immunity genes *CARD4*, *CARD8 *and *CARD15 *are not associated with susceptibility to CRC in German population. In accordance with previous studies, these findings suggest that such variants are unlikey to play a major role disease development in the majority of CRC patients. However, our findings suggest a different situation for CRC patients with early disease manifestation. For this patient subgroup *CARD15 *R702W was found to be significantly associated with disease susceptibility. Independent studies are also needed to examine the effect of *CARD4 *E266K in early onset CRC patients.

## Competing interests

The authors declare that they have no competing interests.

## Authors' contributions

NM, WS, BS, CR, PR, CR, recruited and characterized the patients. SB, OK, AF performed and supervised the data analysis. JE, PR, MB, HK, UF, CB, JT, SH participated in study design and the drafting of the manuscript. MK and SS structured the patient recruitment project. JH and CS conceived and coordinated the study and drafted the manuscript. All authors have read and approved the final manuscript.

## Pre-publication history

The pre-publication history for this paper can be accessed here:

http://www.biomedcentral.com/1471-230X/9/79/prepub

## References

[B1] Hemminki K Chen B Familial risk for colorectal cancers are mainly due to heritable causes Cancer Epidemiol Biomarkers Prev 2004 13 7 1253 1256 15247139

[B2] Andrieu N Launoy G Guillois R Ory-Paoletti C Gignoux M Familial relative risk of colorectal cancer: a population-based study Eur J Cancer 2003 39 13 1904 1911 10.1016/S0959-8049(03)00420-9 12932670

[B3] Johns LE Kee F Collins BJ Patterson CC Houlston RS Colorectal cancer mortality in first-degree relatives of early-onset colorectal cancer cases Dis Colon Rectum 2002 45 5 681 686 10.1007/s10350-004-6267-0 12004220

[B4] Balkwill F Mantovani A Inflammation and cancer: back to Virchow? Lancet 2001 357 9255 539 545 10.1016/S0140-6736(00)04046-0 11229684

[B5] Munkholm P Review article: the incidence and prevalence of colorectal cancer in inflammatory bowel disease Aliment Pharmacol Ther 2003 18 Suppl 2 1 5 10.1046/j.1365-2036.18.s2.2.x 12950413

[B6] Rhodes JM Campbell BJ Inflammation and colorectal cancer: IBD-associated and sporadic cancer compared Trends Mol Med 2002 8 1 10 16 10.1016/S1471-4914(01)02194-3 11796261

[B7] Hugot JP Chamaillard M Zouali H Lesage S Cezard JP Belaiche J Almer S Tysk C O'Morain CA Gassull M Association of NOD2 leucine-rich repeat variants with susceptibility to Crohn's disease Nature 2001 411 6837 599 603 10.1038/35079107 11385576

[B8] Ogura Y Bonen DK Inohara N Nicolae DL Chen FF Ramos R Britton H Moran T Karaliuskas R Duerr RH A frameshift mutation in NOD2 associated with susceptibility to Crohn's disease Nature 2001 411 6837 603 606 10.1038/35079114 11385577

[B9] Hampe J Cuthbert A Croucher PJ Mirza MM Mascheretti S Fisher S Frenzel H King K Hasselmeyer A MacPherson AJ Association between insertion mutation in NOD2 gene and Crohn's disease in German and British populations Lancet 2001 357 9272 1925 1928 10.1016/S0140-6736(00)05063-7 11425413

[B10] Cuthbert AP Fisher SA Mirza MM King K Hampe J Croucher PJ Mascheretti S Sanderson J Forbes A Mansfield J The contribution of NOD2 gene mutations to the risk and site of disease in inflammatory bowel disease Gastroenterology 2002 122 4 867 874 10.1053/gast.2002.32415 11910337

[B11] Vermeire S Wild G Kocher K Cousineau J Dufresne L Bitton A Langelier D Pare P Lapointe G Cohen A CARD15 genetic variation in a Quebec population: prevalence, genotype-phenotype relationship, and haplotype structure Am J Hum Genet 2002 71 1 74 83 10.1086/341124 12019468 PMC384994

[B12] Inohara N Nunez G The NOD: a signaling module that regulates apoptosis and host defense against pathogens Oncogene 2001 20 44 6473 6481 10.1038/sj.onc.1204787 11607846

[B13] McGovern DP Hysi P Ahmad T van Heel DA Moffatt MF Carey A Cookson WO Jewell DP Association between a complex insertion/deletion polymorphism in NOD1 (CARD4) and susceptibility to inflammatory bowel disease Hum Mol Genet 2005 14 10 1245 1250 10.1093/hmg/ddi135 15790594

[B14] McGovern DP Butler H Ahmad T Paolucci M van Heel DA Negoro K Hysi P Ragoussis J Travis SP Cardon LR TUCAN (CARD8) Genetic Variants and Inflammatory Bowel Disease Gastroenterology 2006 131 4 1190 1196 10.1053/j.gastro.2006.08.008 17030188

[B15] Kurzawski G Suchy J Kladny J Grabowska E Mierzejewski M Jakubowska A Debniak T Cybulski C Kowalska E Szych Z The NOD2 3020insC mutation and the risk of colorectal cancer Cancer Res 2004 64 5 1604 1606 10.1158/0008-5472.CAN-03-3791 14996717

[B16] Papaconstantinou I Theodoropoulos G Gazouli M Panoussopoulos D Mantzaris GJ Felekouras E Bramis J Association between mutations in the CARD15/NOD2 gene and colorectal cancer in a Greek population Int J Cancer 2005 114 3 433 435 10.1002/ijc.20747 15578724

[B17] Roberts RL Gearry RB Allington MD Morrin HR Robinson BA Frizelle FA Caspase recruitment domain-containing protein 15 mutations in patients with colorectal cancer Cancer Res 2006 66 5 2532 2535 10.1158/0008-5472.CAN-05-4165 16510569

[B18] Alhopuro P Ahvenainen T Mecklin JP Juhola M Jarvinen HJ Karhu A Aaltonen LA NOD2 3020insC alone is not sufficient for colorectal cancer predisposition Cancer Res 2004 64 20 7245 7247 10.1158/0008-5472.CAN-04-2364 15492242

[B19] Tuupanen S Alhopuro P Mecklin JP Jarvinen H Aaltonen LA No evidence for association of NOD2 R702W and G908R with colorectal cancer Int J Cancer 2007 121 1 76 79 10.1002/ijc.22651 17351900

[B20] Lakatos PL Hitre E Szalay F Zinober K Fuszek P Lakatos L Fischer S Osztovits J Gemela O Veres G Common NOD2/CARD15 variants are not associated with susceptibility or the clinicopathologic characteristics of sporadic colorectal cancer in Hungarian patients BMC Cancer 2007 7 54 10.1186/1471-2407-7-54 17389035 PMC1847447

[B21] Krawczak M Nikolaus S von Eberstein H El Mokhtari NE Schreiber S PopGen: Population-based recruitment of patients and controls for the analysis of complex genotype-phenotype relationships Community Genet 2006 9 1 55 61 10.1159/000090694 16490960

[B22] Rodriguez-Bigas MA Boland CR Hamilton SR Henson DE Jass JR Khan PM Lynch H Perucho M Smyrk T Sobin L A National Cancer Institute Workshop on Hereditary Nonpolyposis Colorectal Cancer Syndrome: meeting highlights and Bethesda guidelines Journal of the National Cancer Institute 1997 89 23 1758 1762 10.1093/jnci/89.23.1758 9392616

[B23] Hampe J Wollstein A Lu T Frevel HJ Will M Manaster C Schreiber S An integrated system for high throughput TaqMan based SNP genotyping Bioinformatics (Oxford, England) 2001 17 7 654 655 10.1093/bioinformatics/17.7.654 11448884

[B24] Hampe J Grebe J Nikolaus S Solberg C Croucher PJ Mascheretti S Jahnsen J Moum B Klump B Krawczak M Association of NOD2 (CARD 15) genotype with clinical course of Crohn's disease: a cohort study Lancet 2002 359 9318 1661 1665 10.1016/S0140-6736(02)08590-2 12020527

[B25] Altshuler D Brooks LD Chakravarti A Collins FS Daly MJ Donnelly P A haplotype map of the human genome Nature 2005 437 7063 1299 1320 10.1038/nature04226 16255080 PMC1880871

[B26] Dudbridge F Pedigree disequilibrium tests for multilocus haplotypes Genetic epidemiology 2003 25 2 115 121 10.1002/gepi.10252 12916020

[B27] Dupont WD Plummer WD PS power and sample size program available for free on the Internet Controlled Clin Trials 1997 18 274 10.1016/S0197-2456(97)00074-3

[B28] de Bakker PI Yelensky R Pe'er I Gabriel SB Daly MJ Altshuler D Efficiency and power in genetic association studies Nature genetics 2005 37 11 1217 1223 10.1038/ng1669 16244653

[B29] Barrett JC Fry B Maller J Daly MJ Haploview: analysis and visualization of LD and haplotype maps Bioinformatics (Oxford, England) 2005 21 2 263 265 10.1093/bioinformatics/bth457 15297300

[B30] Franke A Ruether A Wedemeyer N Karlsen TH Nebel A Schreiber S No association between the functional CARD4 insertion/deletion polymorphism and inflammatory bowel diseases in the German population Gut 2006 55 11 1679 1680 10.1136/gut.2006.104646 17047129 PMC1860122

[B31] Molnar T Hofner P Nagy F Lakatos PL NOD1 gene E266K polymorphism is associated with disease susceptibility but not with disease phenotype or NOD2/CARD15 in Hungarian patients with Crohn's disease Dig Liver Dis 2007 39 1064 1070 10.1016/j.dld.2007.09.003 17964870

[B32] Croucher PJ Mascheretti S Hampe J Huse K Frenzel H Stoll M Lu T Nikolaus S Yang SK Krawczak M Haplotype structure and association to Crohn's disease of CARD15 mutations in two ethnically divergent populations Eur J Hum Genet 2003 11 1 6 16 10.1038/sj.ejhg.5200897 12529700

[B33] Coussens LM Werb Z Inflammation and cancer Nature 2002 420 6917 860 867 10.1038/nature01322 12490959 PMC2803035

[B34] Shacter E Weitzman SA Chronic inflammation and cancer Oncology (Williston Park) 2002 16 2 217 226 discussion 230-212. 11866137

[B35] Yamamoto-Furusho JK Podolsky DK Innate immunity in inflammatory bowel disease World J Gastroenterol 2007 13 42 5577 5580 17948931 10.3748/wjg.v13.i42.5577 PMC4172736

[B36] Ke X Durrant C Morris AP Hunt S Bentley DR Deloukas P Cardon LR Efficiency and consistency of haplotype tagging of dense SNP maps in multiple samples Human molecular genetics 2004 13 21 2557 2565 10.1093/hmg/ddh294 15367493

[B37] Hirschhorn JN Daly MJ Genome-wide association studies for common diseases and complex traits Nat Rev Genet 2005 6 2 95 108 10.1038/nrg1521 15716906

[B38] Helms C Cao L Krueger JG Wijsman EM Chamian F Gordon D Heffernan M Daw JA Robarge J Ott J A putative RUNX1 binding site variant between SLC9A3R1 and NAT9 is associated with susceptibility to psoriasis Nature genetics 2003 35 4 349 356 10.1038/ng1268 14608357

[B39] Tokuhiro S Yamada R Chang X Suzuki A Kochi Y Sawada T Suzuki M Nagasaki M Ohtsuki M Ono M An intronic SNP in a RUNX1 binding site of SLC22A4, encoding an organic cation transporter, is associated with rheumatoid arthritis Nature genetics 2003 35 4 341 348 10.1038/ng1267 14608356

[B40] Robert-Koch-Institut Krebs in Deutschland 2003-2004. Häufigkeiten und Trendsüberarbeitete Auflage. Berlin: Gesellschaft der epidemiologischen Krebsregister in Deutschland e.V 2008 6

[B41] Claus EB Risch NJ Thompson WD Age at onset as an indicator of familial risk of breast cancer American journal of epidemiology 1990 131 6 961 972 10.1002/mds.20663 2188501

[B42] Pankratz N Byder L Halter C Rudolph A Shults CW Conneally PM Foroud T Nichols WC Presence of an APOE4 allele results in significantly earlier onset of Parkinson's disease and a higher risk with dementia Mov Disord 2006 21 1 45 49 10.1002/gepi.20043 16116614

[B43] Pankratz VS de Andrade M Therneau TM Random-effects Cox proportional hazards model: general variance components methods for time-to-event data Genetic epidemiology 2005 28 2 97 109 10.1093/hmg/ddm169 15532036

[B44] van Limbergen J Nimmo ER Russell RK Investigation of NOD1/CARD4 variation in inflammatory bowel disease using a haplotype-tagging strategy Hum Mol Genet 2007 16 18 2175 2186 10.1053/gast.2003.50019 17613538

[B45] Bonen DK Ogura Y Nicolae DL Crohn's Disease-Associated NOD2 Variants Share a Signaling Defect in Response to Lipopolysaccharide and Peptidoglycan Gastroenterology 2003 124 140 146 10.1016/j.micinf.2007.01.015 12512038

[B46] Rosenstiel P Till A Schreiber S NOD-like receptors and human diseases Microbes and Infection 2007 9 648 657 10.1038/ng2085 17376727

[B47] Tomlinson I Webb E Carvajal-Carmona L Broderick P Kemp Z Spain S Penegar S Chandler I Gorman M Wood W A genome-wide association scan of tag SNPs identifies a susceptibility variant for colorectal cancer at 8q24.21 Nat Genet 2007 39 8 984 988 10.1038/ng2089 17618284

[B48] Zanke BW Greenwood CM Rangrej J Kustra R Tenesa A Farrington SM Prendergast J Olschwang S Chiang T Crowdy E Genome-wide association scan identifies a colorectal cancer susceptibility locus on chromosome 8q24 Nat Genet 2007 39 8 989 994 10.1038/ng.111 17618283

[B49] Tomlinson IP Webb E Carvajal-Carmona L Broderick P Howarth K Pittman AM Spain S Lubbe S Walther A Sullivan K A genome-wide association study identifies colorectal cancer susceptibility loci on chromosomes 10p14 and 8q23.3 Nat Genet 2008 40 5 623 630 10.1038/ng.133 18372905

[B50] Tenesa A Farrington SM Prendergast JG Porteous ME Walker M Haq N Barnetson RA Theodoratou E Cetnarskyj R Cartwright N Genome-wide association scan identifies a colorectal cancer susceptibility locus on 11q23 and replicates risk loci at 8q24 and 18q21 Nat Genet 2008 40 5 631 637 10.1038/ng.2007.18 18372901 PMC2778004

[B51] Broderick P Carvajal-Carmona L Pittman AM Webb E Howarth K Rowan A Lubbe S Spain S Sullivan K Fielding S A genome-wide association study shows that common alleles of SMAD7 influence colorectal cancer risk Nat Genet 2007 39 11 1315 1317 10.1038/ng.262 17934461

[B52] Houlston RS Webb E Broderick P Pittman AM Di Bernardo MC Lubbe S Chandler I Vijayakrishnan J Sullivan K Penegar S Meta-analysis of genome-wide association data identifies four new susceptibility loci for colorectal cancer Nat Genet 2008 40 12 1426 1435 10.1038/ng.2007.41 19011631 PMC2836775

[B53] Jaeger E Webb E Howarth K Carvajal-Carmona L Rowan A Broderick P Walther A Spain S Pittman A Kemp Z Common genetic variants at the CRAC1 (HMPS) locus on chromosome 15q13.3 influence colorectal cancer risk Nat Genet 2008 40 1 26 28 10.1002/ijc.23872 18084292

[B54] Schafmayer C Buch S Volzke H von Schonfels W Egberts JH Schniewind B Brosch M Ruether A Franke A Mathiak M Investigation of the colorectal cancer susceptibility region on chromosome 8q24.21 in a large German case-control sample Int J Cancer 2009 124 1 75 80 10.1038/sj.gene.6364111 18839428

[B55] Arnott ID Nimmo ER Drummond HE Fennell J Smith BR MacKinlay E Morecroft J Anderson N Kelleher D O'Sullivan M NOD2/CARD15, TLR4 and CD14 mutations in Scottish and Irish Crohn's disease patients: evidence for genetic heterogeneity within Europe? Genes Immun 2004 5 5 417 425 10.1038/sj.gene.6364111 15190267

